# Cisplatin combined with capecitabine-induced chemotherapy for local nasopharyngeal carcinoma can improve the quality of life and reduce toxic and side effects

**DOI:** 10.1186/s12957-021-02393-1

**Published:** 2021-09-17

**Authors:** Ying Gao, Zhe Liu, Yiting Liu

**Affiliations:** 1 Department of Otorhinolaryngology, Affiliated Hospital of Yan’an University, Yan’an, 716000 Shanxi Province China; 2 Department of Cardiovascular Medicine, Affiliated Hospital of Yan’an University, Yan’an, 716000 Shanxi Province China; 3 Department of Medical Oncology, Affiliated Hospital of Yan’an University, 43 North Street, Baota District, Yan’an, 716000 Shanxi Province China

**Keywords:** Capecitabine, Cisplatin, Nasopharyngeal carcinoma, Toxic and side effects

## Abstract

**Background:**

This study was designed to probe into the effect of cisplatin combined with capecitabine on nasopharyngeal carcinoma (NPC).

**Methods:**

A total of 136 NPC patients treated for the first time in our hospital from January 2016 to March 2017 were collected and divided into two groups: A and B. Among them, 66 in group A were treated with cisplatin intravenous drip, while 70 in group B were treated with capecitabine on the basis of group A. The efficacy, toxic and side effects, and quality of life of the two groups were observed.

**Results:**

The short-term efficacy of group B was better than that of group A (*p*<0.05). The toxic and side effects of group B were lower than that of group A (*p*<0.05). The quality of life in group B was higher than that in group A (*p*<0.05).

**Conclusions:**

Cisplatin combined with capecitabine-induced chemotherapy for local NPC can improve the quality of life and reduce the toxic and side effects.

## Introduction

Among all cancers, the morbidity of nasopharyngeal carcinoma (NPC) is very high in epidemic areas. NPC is a kind of head and neck cancer with a low survival rate, which is rare in most parts of the world, tumor epidemiology in 2018 Statistics show that there were 120,000 new cases of nasopharyngeal cancer in 2018, and 72,000 deaths due to disease, accounting for 0.7% and 0.8% of all tumors [1, 2]. The reason is that it has a unique geographical pattern [3]. Surgical resection, radiotherapy alone, or concurrent chemoradiotherapy are important treatment methods for NPC [4]. However, considering the close distance between nasopharynx and brain stem cells, major blood vessels, and nerves, surgical resection is usually the last choice for advanced and metastatic diseases [5]. For a long time, radiotherapy has been regarded as the main treatment for NPC. However, the further efficacy of radical radiotherapy has reached the bottleneck of advanced patients, who are prone to relapse and distant metastasis after treatment [6, 7]. In addition, induction chemotherapy can also achieve better results, especially in remote control [8]. It may be necessary to face the problem of increased toxicity. If we can customize good adjuvant treatment for NPC patients to avoid unnecessary toxicity, it will be an ideal choice.

Cisplatin is an anti-tumor drug and the most widely used chemotherapy, which can be used as the backbone of various malignancy treatment programs and improve the survival rate and cure rate [9]. However, it is excreted from the kidney and may accumulate in the proximal tubules, resulting in nephrotoxicity [10]. Moreover, many cancers initially respond to platinum therapy, but when the tumor recurs, it often produces drug resistance, which reduces efficacy [11]. Capecitabine is an oral pro-drug of 5-fluorouracil, which can inhibit DNA synthesis. It has been widely used to treat many solid malignancies, especially breast cancer (BC), gastrointestinal cancer (GC), and pancreatic cancer (PC) [12]. Besides, it has advantages over other therapeutic agents, so that it has both oral convenience and good toxicity [13]. A recent research has shown that adding capecitabine to standard chemotherapy seems to improve the disease-free survival and overall survival of triple-negative BC [14], and another study has shown that capecitabine as adjuvant can improve the overall survival of biliary tract cancer resection patients [15]. Nevertheless, its role in NPC is vague at present, so this experiment added adjuvant capecitabine to treat local NPC patients with cisplatin and observed the efficacy.

## Methods

### Clinical data

A total of 136 NPC patients treated for the first time in our hospital from January 2016 to March 2017 were collected and divided into two groups: A and B. Among them, 66 in group A were treated with cisplatin intravenous drip, while 70 in group B were treated with capecitabine based on group A. Inclusion criteria: all patients were confirmed as NPC by pathological examination [16] and staged in stages III–IVa; they were ≥18 years old; Karnofsky performance status (KPS) score of 70 or higher; adequate organ function; complete medical history and physical examination; hematologic and biochemical analyses; and imageological examination like MRI, CT, or PET-CT. Exclusion criteria: patients who received radiotherapy, chemotherapy, and radiotherapy and chemotherapy in the past, and those with other tumors, abnormal hematopoietic function, or chemotherapy contraindications were excluded. Patients and their families were informed and they signed an informed consent form, and this test was approved by the Ethics Committee of our hospital. The trial was performed in accordance with the Declaration of Helsinki.

### Treatment methods

Patients in group A were treated with cisplatin alone: cisplatin (SFDA Approval No. H20056422, Fenghuang Pharmaceutical Co., Ltd., Shandong, China), 20 mg/m^2^, intravenous drip, for 5 days. Those in group B were given oral capecitabine (SFDA Approval No. H20073024, Roche Pharmaceutical Co., Ltd., Shanghai, China) on the basis of group A, 1000 mg/m^2^, twice a day, and they took a rest for 1 week after 2 weeks, and 3 weeks were regarded as a course of treatment. In both groups, 3 weeks were taken as one cycle, 3 cycles in total. In order to prevent hand-foot syndrome, vitamin B6 was given orally at the same time, and the maximum daily dose could reach 200 mg.

### Outcome measures

The short-term efficacy of the two groups 1 month after treatment was observed by local measurable lesions: complete remission (CR): all the lesions disappeared and lasted for 1 month; partial remission (PR): the product of the two largest vertical diameters of the tumor was reduced by at least 50%; stable disease (SD): between PR and PD; progressive disease (PD): the product of two maximum vertical diameters of the tumor increased by more than 25%; effective rate = (CR+PR) cases/total cases×100%. The patients’ quality of life after chemotherapy was evaluated by KPS score [17]. The score was out of 100, and it was directly proportional to the quality of life. The survival time was from the beginning of treatment to death or the last follow-up. The 3-year survival, recurrence, and distant metastasis rates were observed after treatment. Altogether, the 5-ml venous blood was collected before and after treatment. Next, 5 ml was centrifuged in a centrifuge (10×*g* at 4°C for 15 min, Beijing BMH Instruments Co., Ltd.), and then, the upper serum was drawn. The serum IL-12 (interleukin-12), matrix metalloproteinase-2 (MMP-2), and IFN-γ (interferon-γ) levels were tested by ELISA (Suzhou ELSBIO Biotechnology Co., Ltd.) in view of the instructions.

### Statistical methods

SPSS 21.0 (SPSS, Inc., Chicago, IL, USA) was used for statistical analysis. The measurement data were expressed by (*x*±sd). Those between groups were compared by *T* test, and those before and after treatments were assessed by paired *T* test. The counting data were expressed by [*n*(%)], and those between groups were compared by chi-square test. The KM survival curve was used to plot the overall survival of patients, and the log-rank test was used for analysis. The difference was statistically remarkable when *p*<0.05.

## Results

### General data of patients in both groups

In this study, we compared the clinical data of the two groups of patients. Through comparison, we found that there were no statistical differences in gender, age, ethnicity, T stage, N stage, clinical stage, and pathological type between the two groups (Table 1, *p*>0.05)

**Table 1:** Comparison of general data between both groups (*x*±sd) [*n*(%)]

Classification	Group A (*n*=66)	Group B (*n*=70)	*t*/*χ*^2^ value	*p* value
Gender			0.471	0.492
Male	31 (46.97)	37 (52.86)		
Female	35 (53.03)	33 (47.14)		
Age (years)	46.13±5.18	47.11±5.12	1.109	0.269
Nationality			0.737	0.390
Han	54 (81.82)	61 (87.14)		
Ethnic minorities	12 (18.18)	9 (12.86)		
T staging			0.377	0.828
T1	0 (0.00)	0 (0.00)		
T2	19 (28.79)	17 (24.28)		
T3	22 (33.33)	24 (34.29)		
T4	25 (37.88)	29 (41.43)		
*N* staging			0.430	0.933
N0	9 (13.64)	11 (15.71)		
N1	27 (40.91)	25 (35.71)		
N2	18 (27.27)	21 (30.00)		
N3	12 (18.18)	13 (18.57)		
Clinical staging			0.047	0.826
Stage III	28 (42.42)	31 (44.29)		
Stage IVa	38 (57.58)	39 (55.71)		
Pathological types			1.550	0.460
Low differentiated squamous cell carcinoma	36 (54.55)	40 (57.14)		
Undifferentiated carcinoma	28 (42.42)	25 (35.71)		
Adenocarcinoma	2 (3.03)	5 (7.14)		

.

### Comparison of a short-term efficacy between the two groups

In order to determine the improvement effect of different treatment options on the patient’s condition, we evaluated the clinical efficacy of the two groups of patients after treatment through the RECIST 1.1 standard. The results showed that after treatment in group A, there were 21 CR patients, 19 PR patients, 12 SD patients, and 14 PD patients. The total effective rate was 60.61%. After treatment in group B, there were 34 CR patients, 23 PR patients, 7 SD patients, and 6 PD patients. The total effective rate was 81.43%. Chi-square test showed that the total effective rate of group B patients was significantly higher than that of group A (Table 2, *p*<0.05). It shows that cisplatin combined with capecitabine induction chemotherapy can improve the clinical efficacy of patients.

**Table 2:** Comparison of short-term efficacy between both groups [*n*(%)]

Efficacy	Group A (*n*=66)	Group B (*n*=70)	*χ*^2^ value	*p* value
CR	21 (31.82)	34 (48.57)	-	-
PR	19 (28.79)	23 (32.86)	-	-
SD	12 (18.18)	7 (10.00)	-	-
PD	14 (21.21)	6 (8.57)	-	-
Total effective rate	40 (60.61)	57 (81.43)	7.201	0.007

### KPS scores of the two groups before and after treatment

**Fig. 1:**
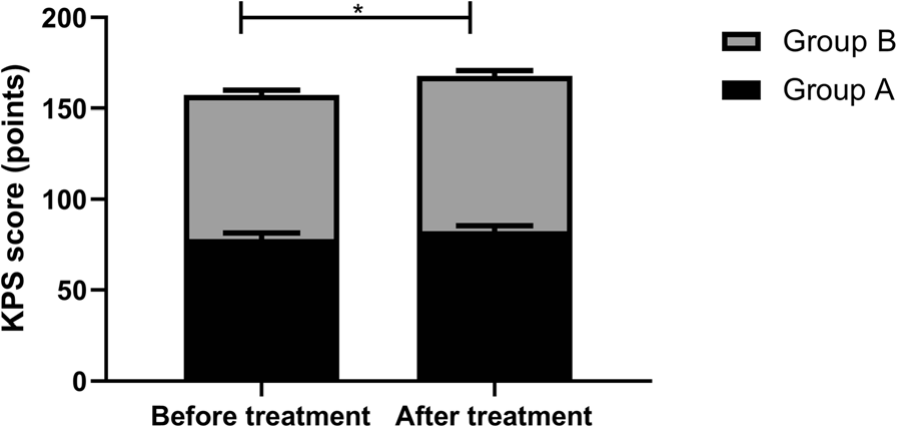
KPS scores of both groups before and after treatment. There is no difference in KPS scores between the two groups before treatment, but after treatment, the scores are improved and those of group B are higher than those of group A (*p*<0.05). Note: ^*^p<0.05

In this study, we also compared the changes in the KPS scores of the two groups of patients. By comparison, we found that there was no statistical difference in the KPS scores of the two groups of patients before treatment (*p*>0.05). After treatment, the KPS score of the two groups of patients was significantly lower than that before the treatment, and the KPS score of group B patients was significantly lower than that of group A patients (Fig. 1, *p*<0.05). This shows that cisplatin combined with capecitabine induction chemotherapy can improve the quality of life of patients.

### Comparison of survival, recurrence, and metastasis rates between the two groups after 3 years of treatment

In this study, two groups of patients were followed up for 3 years. During the follow-up period, patients were lost to follow-up. The follow-up rate was 100.00%. During the follow-up period, we made statistics on the survival, recurrence, and metastasis of the patients. Through analysis, we found that the 3-year survival rate of patients in group B was significantly higher than that of patients in group A (*p*<0.05), and the recurrence rate and metastasis rate of patients in group B were significantly lower than those in group A (Table 3, *p*<0.05). This shows that cisplatin combined with capecitabine induction chemotherapy can improve the survival rate of patients and reduce the probability of recurrence and metastasis in patients.

**Table 3:** Comparison of survival, recurrence, and metastasis rates between the two groups after 3 years of treatment [*n*(%)]

Category	Group A (*n*=66)	Group B (*n*=70)	*χ*^2^ value	*p* value
Survival rate	47 (77.27)	60 (85.71)	4.259	0.039
Recurrence rate	13 (19.70)	5 (7.14)	4.662	0.030
Metastasis rate	15 (22.73)	7 (10.00)	4.058	0.044

### Comparison of toxic and side effects between the two groups

In this study, we also compared the toxic and side effects that occurred during the treatment of patients. Through comparison, we found that the total incidence of side effects in group B (10.00%) was significantly lower than that in group A (24.24%) (Table 4, *p*<0.05). This shows that cisplatin combined with capecitabine induction chemotherapy will not increase the occurrence of toxic and side effects in patients.

**Table 4:** Comparison of toxic and side effects between the two groups [*n*(%)]

Toxic and side effects	Group A (*n*=66)	Group B (*n*=70)	*χ*^2^ value	*p* value
Nausea and vomiting	4 (6.06)	3 (4.29)	–	–
Skin reaction	1 (1.52)	0 (0.00)	–	–
Oral mucosa reaction	3 (4.55)	0 (0.00)	–	–
Diarrhea	1 (1.52)	2 (2.86)	–	–
Hand-foot syndrome	2 (3.03)	1 (1.43)	–	–
Peripheral neurotoxicity	3 (4.55)	1 (1.43)	–	–
Urea nitrogen	2 (3.03)	0 (0.00)	–	–
Total reaction rate	16 (24.24)	7 (10.00)	4.904	0.026

### Serum indexes of the two groups before and after treatment

**Fig. 2:**
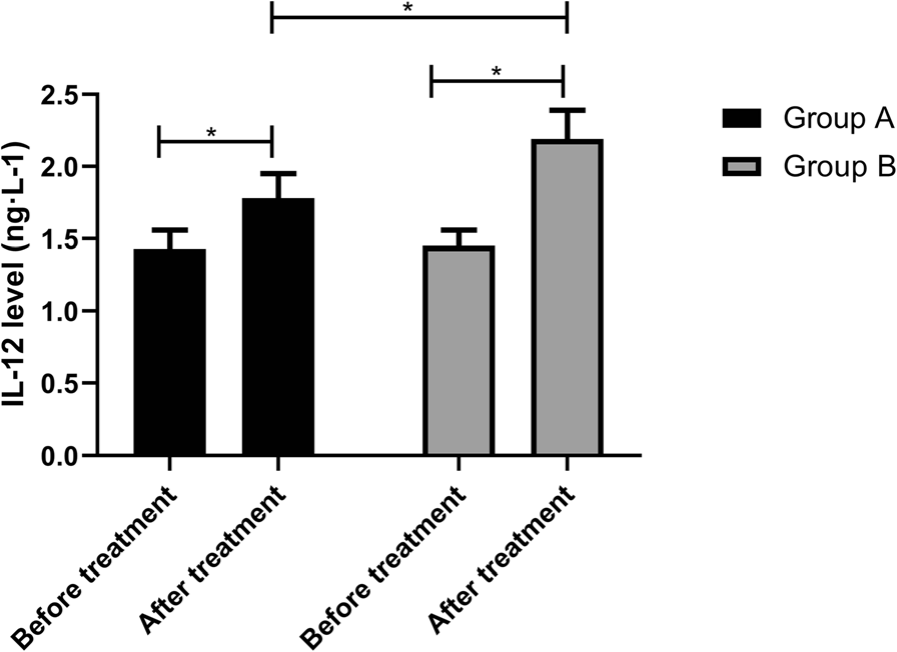
IL-12 levels of both groups before and after treatment. There is no difference in the IL-12 levels between the two groups before treatment, but the levels in group B increased markedly after treatment, and those in group B were higher than those in group A (*p*<0.05). Note: ^*^p<0.05

**Fig. 3:**
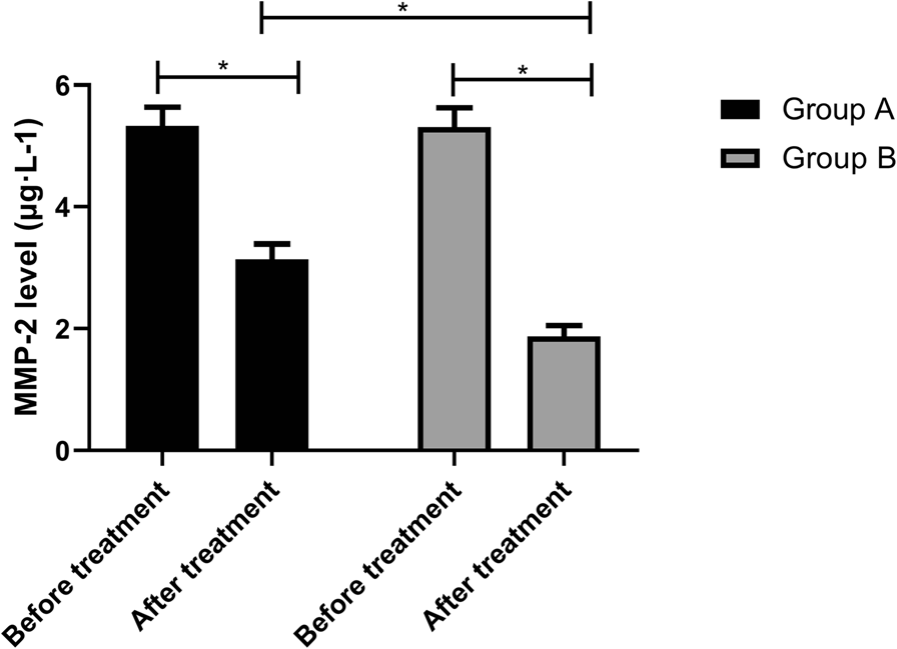
MMP-2 levels before and after treatment in both groups. There is no difference in the MMP-2 levels between the two groups before treatment, but the IL-12 levels decrease markedly after treatment, and the MMP-2 levels in group B are lower than that in group A (*p*<0.05). Note: ^*^p<0.05

**Fig. 4:**
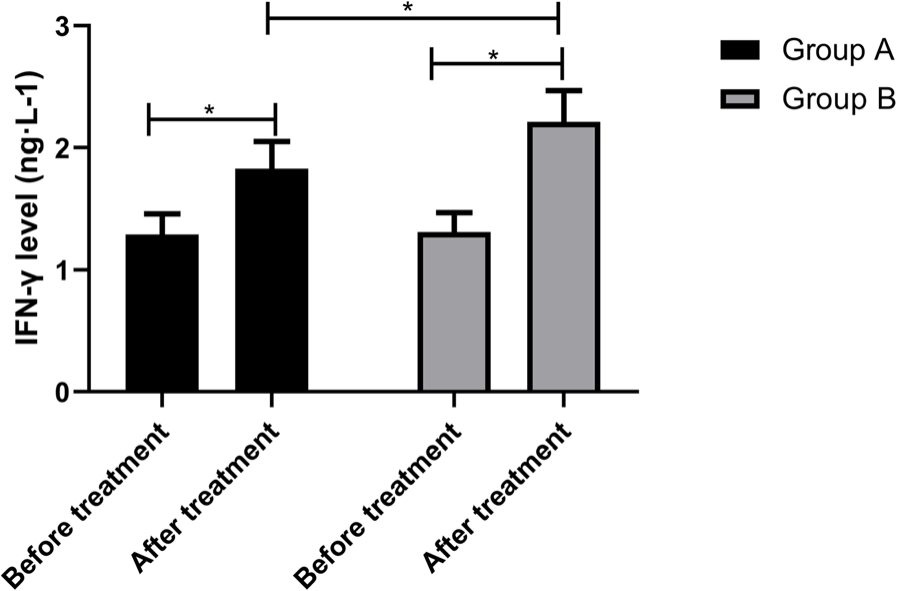
IFN-γ levels of both groups before and after treatment. There is no difference in the IFN-γ levels between the two groups before treatment, but the levels increased remarkably after treatment, and those in group B were higher than those in group A (*p*<0.05). Note: ^*^p<0.05

The IL-12 levels in group A before and after treatment were (1.43±0.13) ng·L-1 and (1.78±0.17) ng·L-1, while those in group B were (1.45±0.11) ng·L-1 and (2.19±0.20) ng·L-1, respectively. The results manifested that the IL-12 levels in group B were higher than those in group A after treatment (*p*<0.05) (Fig. 2). The MMP-2 levels in group A before and after treatment were (5.33±0.31) μg·L-1 and (3.14±0.25) μg·L-1, respectively, while those in group B were (5.31±0.32) μg·L-1 and (1.87±0.18) μg·L-1, respectively. It signified that the MMP-2 levels in group B were lower than those in group A (*p*<0.05) (Fig. 3). The IFN-γ levels in group A before and after treatment were (1.29±0.17) ng·L-1 and (1.83±0.22) ng·L-1, while those in group B were (1.31±0.16) ng·L-1 and (2.21±0.26) ng·L-1, respectively. After treatment, the IFN-γ levels in group B were higher than those in group A (Fig. 4, *p*<0.05).

### Comparison of the quality of life between the two groups after treatment

At the end of the study, we evaluated the quality of life of the patients after treatment. Through evaluation, we found that the cognitive, emotional, overall, and social functions of patients in group B were higher than those in group A (Table 5, *p*<0.05), indicating that the combined treatment had no effect on the quality of life of patients.

**Table 5:** Comparison of quality of life between both groups after treatment (*x*±sd)

Group	*n*	Cognitive function	Emotional function	Overall function	Social function
Group A	66	64.28±4.14	72.11±5.22	70.18±4.92	76.24±6.23
Group B	70	71.34±3.85	77.64±6.27	82.47±4.65	84.11±5.33
*t*		10.300	5.572	14.980	7.930
*p*		<0.001	<0.001	<0.001	<0.001

## Discussion

NPC is a malignant head and neck cancer with apparent regional polymerization. With the development of intensity-modulated radiotherapy and combined chemotherapy, great progress has been made in local and regional control of NPC [18, 19]. Although the 5-year local control rate of NPC has reached from 80 to 90%, 15% to 30% of patients still have distant metastasis [20]. The possible reason for this result is that nearly 70% of the patients were diagnosed with locally advanced diseases when they received treatment [21]. Generally speaking, the combination of cisplatin and 5-fluorouracil has been considered as one of the standard protocols of concurrent radiotherapy and chemotherapy, but the adverse reactions of 5-fluorouracil are cumulative complications of radiotherapy or myelosuppression, which may lead to hospitalization or death related to treatment, thus impairing patients’ quality of life and compliance with treatment [22]. Capecitabine is an oral fluoropyrimidine carbamate, which can be metabolized to fluorouracil by a three-step enzymatic reaction. It has replaced fluorouracil in many chemotherapy regimens for patients with various gastrointestinal cancers. Some experiments have proved that metastatic colorectal cancer patients have good tolerance to capecitabine and have the same anti-tumor activity as fluorouracil [23]. There are also studies showing that capecitabine is used to induce or treat locally advanced head and neck cancer simultaneously, which also shows encouraging results [24].

In this experiment, we observed the short-term efficacy after treatment. It revealed that the total effective rate of cisplatin combined with capecitabine in group A was obviously higher than that of cisplatin alone, which showed that the combination therapy was effective in treating local NPC. Then, we observed and compared the toxic and side effects of the two groups. The results showed that the total incidence of toxic and side effects of cisplatin combined with capecitabine was lower, and the effects were the most common mild adverse events. Studies have shown that patients treated with capecitabine have a good prognosis, good adverse reactions, and no grade 3 to 4 toxicity. What is more, capecitabine seems to be effective and the side effects can be controlled when radiotherapy is carried out simultaneously, which is basically consistent with previous research conclusions, and the two have been mutually verified [25]. All these indicate that cisplatin combined with capecitabine is safe and effective. This may also be because the concentration of capecitabine in tumor cells is much higher than that in normal cells, so it has high anti-tumor activity and low toxicity [26].

The balance of pro-inflammatory cytokine IL-12 plays a key role in shaping the development of anti-tumor or tumor immunity [27], and the anti-tumor activity of IL-12 can be effectively induced by itself and can be markedly improved by combining with various treatments [28]. Matrix metalloproteinases (MMPs) are emerging as the key micro-agents of tissue homeostasis and cell function in various pathologies [29]. However, MMP-2, as one of them, is highly expressed in various pathologies, which interferes with tissue remodeling and inflammatory response. Thus, it is generally believed that blocking the activity of MMP-2 will produce a therapeutic effect [30]. IFN-γ is a key factor driving cellular immunity, which can coordinate various protective functions to enhance the immune response to infection and cancer. It can play its immunomodulatory role by enhancing antigen processing and presentation, increasing leukocyte transportation, inducing antiviral state, enhancing antimicrobial function and affecting cell proliferation and apoptosis [31]. In this experiment, after treatment, the IL-12 and IFN-γ levels in group B were higher, while the MMP-2 levels were lower. It is suggested that cisplatin combined with capecitabine may achieve a therapeutic effect by regulating the level of related factors, but the specific mechanism is still unclear. KPS score is one of the tools that can be used to monitor the changes of vitality and dependence level [32], and it is also a reliable method to measure the performance status of patients [33]. In this experiment, the quality of life of patients after combined treatment is better. This may be because the patients’ confidence in treatment and compliance with treatment are improved after the tumor is controlled accordingly, thus improving negative emotions, improving sleep quality, achieving a good cycle, and further improving their quality of life and quality of life. Finally, we observed the survival rate of patients in both groups after 3 years of treatment. The results manifested that the patients treated with cisplatin combined with capecitabine had higher survival rates and lower recurrence and metastasis rates. There are also studies showing that capecitabine and cisplatin can tolerate adjuvant chemotherapy in D2 resected GC and have advantages in preventing recurrence [34].

In this study, we determined through analysis that cisplatin combined with capecitabine induction chemotherapy can improve the quality of life and side effects of patients with nasopharyngeal carcinoma. However, this study still has certain limitations. First of all, in this experiment, we are not conducting a randomized controlled large-sample study, and the data analyzed and analyzed may be biased. Secondly, in this study, the follow-up time was short and the patients were not followed up for a long time. It is still unclear whether the two drugs have an effect on the long-term survival of patients. Therefore, we hope to collect more samples and conduct long-term follow-up in the follow-up study to improve our research conclusions.

## Conclusions

To sum up, cisplatin combined with capecitabine-induced chemotherapy for local NPC can improve the quality of life and reduce toxic and side effects.

## Data Availability

The datasets used and/or analyzed during the current study are available from the corresponding author on reasonable request.
